# How many general and inflammatory variables need to be fulfilled when defining sepsis due to the 2003 SCCM/ESICM/ACCP/ATS/SIS definitions in critically ill surgical patients: a retrospective observational study

**DOI:** 10.1186/1471-2253-10-22

**Published:** 2010-12-21

**Authors:** Manfred Weiss, Markus Huber-Lang, Michael Taenzer, Martina Kron, Birgit Hay, Maximilian Nass, Moritz Huber, Marion Schneider

**Affiliations:** 1 Department of Anaesthesiology, University Hospital Medical School Ulm, Steinhoevelstr. 9, 89075 Ulm, Germany; 2 Department of Traumatology, Hand-, Plastic-, and Reconstructive Surgery, University Hospital Medical School Ulm, Steinhoevelstr. 9, 89075 Ulm, Germany; 3 Institute of Epidemiology and Medical Biometry, University of Ulm, Schwabstr. 13, 89075 Ulm, Germany; 4 Department of Experimental Anaesthesiology, University Hospital Medical School Ulm, Steinhoevelstr. 9, 89075 Ulm, Germany

## Abstract

**Background:**

It has never been specified how many of the extended general and inflammatory variables of the 2003 SCCM/ESICM/ACCP/ATS/SIS consensus sepsis definitions are mandatory to define sepsis.

**Objectives:**

To find out how many of these variables are needed to identify almost all patients with septic shock.

**Methods:**

Retrospective observational single-centre study in postoperative/posttraumatic patients admitted to an University adult ICU. The survey looked at 1355 admissions, from 01/2007 to 12/2008, that were monitored daily computer-assisted for the eight general and inflammatory variables temperature, heart rate, respiratory rate, significant edema, positive fluid balance, hyperglycemia, white blood cell count and C-reactive protein. A total of 507 patients with infections were classified based on the first day with the highest diagnostic category of sepsis during their stay using a cut-off of 1/8 variables compared with the corresponding classification based on a cut-off of 2, 3, 4, 5, 6, 7 or 8/8 variables.

**Results:**

Applying cut-offs of 1/8 up to 8/8 variables resulted in a decreased detection rate of cases with septic shock, i.e., from 106, 105, 103, 93, 65, 21, 3 to 0. The mortality rate increased up to a cut-off of 6/8 variables, i.e., 31% (33/106), 31% (33/105), 31% (32/103), 32% (30/93), 38% (25/65), 43% (9/21), 33% (1/3) and 0% (0/0).

**Conclusions:**

Frequencies and mortality rates of diagnostic categories of sepsis differ depending on the cut-off for general and inflammatory variables. A cut-off of 3/8 variables is needed to identify almost all patients with septic shock who may benefit from optimal treatment.

## Background

To compare sepsis studies, uniform inclusion criteria are an unequivocal prerequisite. However, no standards for inclusion criteria have been used in sepsis studies prior to 1987 [1]. After the 1992 American College of Chest Physicians/Society of Critical Care Medicine (ACCP/SCCM) consensus conference on sepsis definitions [2], predefined sepsis criteria and markers of organ dysfunction have been increasingly applied for patient enrolment in clinical trials [1]. In the majority of cases since 1993, these ACCP/SCCM definitions have been used as inclusion criteria [1]. A drawback of the 1992 sepsis definitions has been that only four criteria for the detection of a systemic inflammatory response syndrome (SIRS), i.e., temperature, heart rate, respiratory rate, and white blood cell count (WBC), are proposed, with SIRS manifestation being defined as presence of two or more of these four criteria. The other evident disadvantage in the 1992 ACCP/SCCM definitions is that severe sepsis is defined as sepsis plus organ dysfunction, hypoperfusion or hypotension. However, besides lactic acidosis and oliguria, range limits for organ dysfunction variables are missing in the 1992 publication [2].

In 2003, the revised Society of Critical Care Medicine/European Society of Critical Care Medicine/American College of Chest Physicians/American Thoracic Society, Surgical Infection Society (SCCM/ESICM/ACCP/ATS/SIS) sepsis definitions were published to better reflect the reality at the bedside, especially, to address how physicians diagnose sepsis in daily practice regarding general, inflammatory, hemodynamic, organ dysfunction, and tissue perfusion variables [3]. Limits changed from the 1992 definitions to the 2003 definitions, e.g. for fever (from > 38.0°C to > 38.3°C) and tachypnea (from > 20 to > 30 breaths/min). Moreover, an extended list of possible signs of the systemic response was included in the 2003 definitions. In addition, clear cut-offs for organ dysfunctions were provided. However, despite these improvements, the 2003 definitions are scarcely used due to complexity and uncertainty, since the definitions leave unclear how many of the general and inflammatory variables should be used as diagnostic criteria for sepsis.

The diagnosis of severe sepsis and septic shock leads to extensive consequences regarding critical care management and treatment guidelines [4,5]. However, it has not been specified how many of the eleven general and inflammatory variables of the extended list in the 2003 definitions should be fulfilled to define sepsis. Therefore, the present study was performed to find out the frequencies of diagnostic categories of sepsis (sepsis, severe sepsis, septic shock) and ICU mortality rates within the same collective of critically ill postoperative/posttraumatic patients applying the 2003 definitions with increasing cut-offs for the general and inflammatory variables. Moreover, it should be addressed at which cut-off a profound number of patients with septic shock might be under-classified who are expected to benefit from earlier and more focused critical care management.

## Methods

### Patients and data collection

A retrospective observational single-centre study in postoperative/posttraumatic patients admitted to an University adult ICU has been performed. From 01/2007 to 12/2008, all admissions were surveyed daily computer-assisted regarding eight general and inflammatory variables, i.e., temperature, heart rate, respiratory rate, significant edema or positive fluid balance, hyperglycemia, white blood cell count and C-reactive protein. The study is in compliance with the Helsinki declaration and was approved by the Independent Ethics Committee of the University Ulm, which waived informed consent because this was an observational study, and no additional interventions were performed. Patients were admitted to the Anaesthesiology ICU of the University Hospital Ulm after major trauma, great vessel, lung, brain or abdominal surgery. All surgical patients admitted to this ICU were routinely computer-assisted surveyed for presence of sepsis, disease severity (Simplified Acute Physiology Score (SAPS) II) [6] and organ dysfunctions (Sequential Organ Failure Assessment (SOFA) score) [7] on a daily basis by trained ICU residents and staff physicians. To guarantee accuracy and reliability, and to minimize inconsistencies in medical chart reviews, all residents and staff physicians were trained in management of the charts before initiation of the study. After this training phase, these physicians entered score relevant organ function parameters and infection parameters daily during their routine care of the patients in a standardized electronic case report form. Directly after complete data entry of the parameters for the different organ systems and infection, the scores were calculated and displayed. Thereby, the physicians directly received the results of the actual scores and the sepsis classification. A longitudinal overview of the scores, in addition to the actual daily scores, regarding the whole ICU course was presented daily to the residents and staff physicians. They checked, corrected, ascertained and re-ascertained the data. Thus, the severity of disease and organ dysfunction scores, and the diagnostic categories of sepsis classifications (sepsis, severe sepsis, septic shock) were verified. Staff physicians corrected the charts before demission of the patients from the ICU and before final saving in the database. In the present study, data of postoperative/posttraumatic patients admitted to our ICU over a two year period from 01-JAN-2007 until 31-DEC-2008 were analysed. Classification based on the first day with the highest diagnostic category of sepsis using a cut-off of 1/8 variables was compared with the corresponding classification based on a cut-off of 2, 3, 4, 5, 6, 7 or 8/8 variables for each of the patients presented with infections. Only patients ≥ 18 years of age were selected for the present evaluation because SAPS II score [6] and the 2003 SCCM/ESICM/ACCP/ATS/SIS sepsis definitions have been developed for patients ≥ 18 years, and the SOFA score [7] for patients ≥ 12 years. Neurosurgical patients were excluded from analysis because noradrenaline is often used to achieve an adequate cerebral perfusion pressure and not due to shock associated with accompanying infections and sepsis. Since the new diagnostic criteria only refer to the diagnosis of sepsis [3], we focused on patients with sepsis. Thus, patients only revealing SIRS during their stay on the ICU were excluded from analysis.

### Definitions

Sepsis was defined using the 2003 SCCM/ESICM/ACCP/ATS/SIS sepsis definitions [3]. In Table 1 the diagnostic criteria for sepsis and organ dysfunction variables following the 2003 definitions and those used in the present study are specified. If 1/8 up to 8/8 of the general and inflammatory variables in Table 1 were present together with a documented infection, patients were assigned as sepsis patients (Table 1). These eight variables include the general variables temperature, heart rate, respiratory rate, altered mental status (not applied in the present study), significant edema, positive fluid balance, hyperglycemia, and the inflammatory variables white blood cell count (WBC), plasma C-reactive protein, and plasma procalcitonin (not applied in the present study). Altered mental status was not applied in the present study due to the difficulties in the judgement of analgosedated or intubated patients. Plasma procalcitonin was not taken into account, because it was not measured routinely in our patients. Hemodynamic variables were not regarded as diagnostic criteria for sepsis, since the definition of arterial hypotension overlaps with the classification of septic shock, and mixed venous oxygen saturation (SvO_2_) nor cardiac index was measured routinely in all patients. Severe sepsis was defined as sepsis plus organ dysfunction [3]. Organ dysfunctions were defined according to the limitations for organ dysfunction variables and tissue perfusion variables (hyperlactatemia) as given in the original publication [3] and presented in Table 1. Septic shock was defined as sepsis plus shock [3]. Septic shock was defined as hypotension despite adequate volume resuscitation, a systolic blood pressure of ≤ 90 mmHg, or the need of vasopressors to keep blood pressure ≥ 90 mmHg.

**Table 1:** Diagnostic criteria and organ dysfunction variables within the 2003 SCCM/ESICM/ACCP/ATS/SIS sepsis definitions

	SCCM/ESICM/ACCP/ ATS/SIS 2003 original publication	SCCM/ESICM/ACCP/ ATS/SIS 2003 used in present study
**Diagnostic criteria for sepsis**		
**Infection**	+	+
**General variables**		
Temperature	< 36.0°C and/or >38.3°C	< 36.0°C and/or >38.3°C
Heart rate	> 90	> 90
Respiratory rate	> 30	> 30
Altered mental status	+	n. a.
Significant edema	+	+
Positive fluid balance	> 20 ml/kg over 24 hrs	> 20 ml/kg over 24 hrs
Hyperglycemia in absence of diabetes	> 120 mg/dl or 7.7 mmol/l	> 120 mg/dl or 7.7 mmol/l
**Inflammatory variables**		
White blood cell count (WBC)	> 12,000/μl and/or < 4,000/μl and/or > 10% immature	> 12,000/μl and/or < 4,000/μl and/or (> 10% immature, n. a.)
Plasma C-reactive protein	> 2 SD above normal	> 4 mg/l
Plasma procalcitonin	> 2 SD above normal	n. a.
**Hemodynamic variables**		
Arterial hypotension	SBP < 90 mmHg and/or MAP < 70 mmHg and/or SBP decrease > 40 mmHg	n. a.
SvO_2_	> 70%	n. a.
Cardiac index	> 3.5 l/min/m^2^	n. a.
**Organ dysfunction variables**		
Arterial hypoxemia	PaO_2_/FiO_2 _< 300	PaO_2_/FiO_2 _< 300
Acute oliguria	< 0.5 ml/kg/h or 45 mmol/l >2 h	< 0.5 ml/kg/h or 45 mmol/l >2 h
Hypoperfusion or hypotension	+	n. a.
Creatinin increase	> 0.5 mg/dl or 43 μmol/l	> 0.5 mg/dl or 43 μmol/l
Coagulation abnormalities	INR >1.5 or aPTT >60 s	INR >1.5 or aPTT >60 s
Ileus (absent bowel sounds)	+	+
Thrombocytopenia, platelet count	< 100,000/μl	< 100,000/μl
Hyperbilirubinemia, plasma total bilirubin	> 4 mg/dl or 70 μmol/l	> 4 mg/dl or 70 μmol/l
**Tissue perfusion variables**		
Hyperlactatemia	> 1 mmol/l	> 1 mmol/l

n. a. = not applied, INR = International Normalized Ratio, MAP = mean arterial pressure, PaO_2_/FiO_2 _= partial pressure of arterial oxygen/fraction of inspired oxygen, SBP = systolic blood pressure, SOFA = Sequential Organ Failure Assessement, SvO_2 _= mixed venous oxygen saturation.

The classification result, for each patient, based on the first day with the highest diagnostic category of sepsis (sepsis, severe sepsis, septic shock) defined with a cut-off of one out of the eight general and inflammatory variables during his/her ICU stay was compared with the corresponding classification results based on a cut-off of 2, 3, 4, 5, 6, 7, or 8/8 variables.

### Statistical analyses

The frequencies of the diagnostic categories sepsis, severe sepsis and septic shock were determined according to the classification using 1/8 up to 8/8 general and inflammatory variables as a cut-off to classify sepsis, and ICU mortality rates among patients with septic shock were compared descriptively. The degree of agreement between the classifications using 1/8 up to 8/8 general and inflammatory variables to classify sepsis was estimated using the Kappa coefficient. Odds ratios (ORs) for a fatal outcome among patients with septic shock compared to those without septic shock were calculated using a cut-off of 1/8 up to 8/8 variables. Odds ratios (ORs) are presented with corresponding 95% confidence interval (CI).

## Results

A total of 1628 postoperative/posttraumatic patients were admitted from 01 Jan 2007 to 31 Dec 2008 in the ICU and were surveyed daily using computer-assistance with respect to sepsis, organ dysfunctions assessment and shock based on the 2003 SCCM/ESICM/ACCP/ATS/SIS sepsis definitions [3]. 1355 patients ≥ 18 years of age with a total of 8955 observations were available.

Out of the 1355 cases, 366 cases were admitted to our ICU after abdominal surgery, 324 cases after great vessel or lung surgery, 235 cases after major trauma and damage control orthopaedic surgery, 428 cases due to neurosurgery and 2 cases due to other reasons. After exclusion of the nonrelevant cases, 507 cases remained being classified as sepsis patients. The main causes of infections were pneumonia, bloodstream infections, intravascular catheter-related infections, intra-abdominal infections, urological infections and surgical wound infections. Clinical characteristics of the 507 cases were as follows. Median age was 68 years (range: 18 to 98 years; mean +/- SD: 64 +/- 15 years). 156 of 507 cases were female and 351 were male. Median SAPS II was 38 (range: 4 to 97; mean +/- SD: 40 +/- 17). Median SOFA score (due to analgosedation without Glasgow Coma Scale, thus, theoretical maximum of 20) was 8 (range: 0 to 18; mean +/- SD: 8 +/- 3).

**Figure 1:**
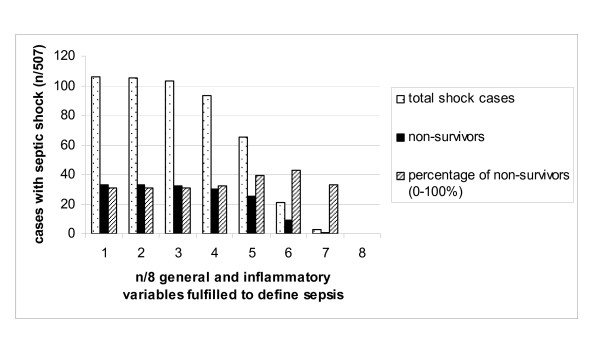
**Cases with septic shock and corresponding mortality rate in 507 cases applying a cut-off of 1/8 up to 8/8 general and inflammatory variables to define sepsis**.

Within this patient collective (n = 507), applying a cut-off of 1/8 up to 8/8 general and inflammatory variables resulted in a decrease of cases classified as septic shock with increasing mortality rates (Figure 1; Table 2). The odds ratio (OR) of death of septic shock patients compared to those not classified as shock patients applying a cut-off of 1/8 up to 7/8 variables increased with 95% confidence intervals overlapping (Table 2).

**Table 2:** Mortality rate in cases with septic shock applying a cut-off of 1/8 up to 8/8 general or inflammatory variables to define sepsis.

x/8 general/inflammatory criteria fulfilled	Cases in septic shock	Non-survivors of septic shock cases		Risk of death of septic shock patients	
	n	n	(%)	OR	95% CI
1/8	106	33	31%	3.2	2.0 - 5.4
2/8	105	33	31%	3.3	2.0 - 5.5
3/8	103	32	31%	3.2	1.9 - 5.3
4/8	93	30	32%	3.3	2.0 - 5.6
5/8	65	25	38%	4.2	2.4 - 7.5
6/8	21	9	43%	4.2	1.7 - 10.4
7/8	3	1	33%	2.6	0.2 - 29.1
8/8	0	0	0%	0	0

Moreover, the risk of death in cases classified as septic shock (OR + 95% CI) compared to the cases not classified as shock patients is given.

The agreement between the classification of septic shock using 1/8 vs. 2/8 up to 7/8 vs. 8/8 criteria was estimated using the Kappa coefficient (Table 3). Comparing the number of cases with septic shock between the classifications using 1/8 vs. 2/8 up to 7/8 vs. 8/8 criteria, disagreement cumulated to 106 cases, i.e., 21% in total. Overall, more than 8% of septic shock cases classified by the definition using a cut-off of 1/8 were under-classified when using a cut-off of > 4/8 variables to assign sepsis.

**Table 3:** Disagreement of classifications of septic shock, if sepsis is defined with 1/8 vs. up to 8/8 general and inflammatory variables.

x/8 general inflammatory criteria fulfilled	Septic shock cases with no agreement		Kappa coefficient	Sum of septic shock cases with no agreement	
	x	(% of 507)		n	(% of 507)
1/8 vs. 2/8	1	0%	0.9940	1	0%
2/8 vs. 3/8	2	0%	0.9879	3	1%
3/8 vs. 4/8	10	2%	0.9368	13	3%
4/8 vs. 5/8	28	6%	0.7913	41	8%
5/8 vs. 6/8	44	9%	0.4542	85	17%
6/8 vs. 7/8	18	4%	0.2422	103	20%
7/8 vs. 8/8	3	1%	0	106	21%

## Discussion

The present study shows that defining sepsis with cut-offs at 1, 2, 3, 4, 5, 6, 7, or 8 out of 8 general and inflammatory variables was markedly associated with frequency and mortality rate of cases with septic shock in critically ill surgical patients. Frequency of septic shock continuously decreased for cut-offs from 1/8 up to 8/8 criteria, while mortality increased for cut-offs from 1/8 up to 6/8. Only one septic shock case was lost when changing the cut-off from 1/2 to 2/8 variables. However, a change in the cut-offs from 3/8 to 4/8 criteria resulted in a profound decrease of cases in septic shock from 103 to 93 cases. Summed up, 13 out of 106 septic shock cases, i.e., 12%, who potentially might benefit from earlier and more focused critical care management would not have been detected when changing the cut-off from 1/8 to 4/8 variables. Thus, we suggest to set the cut-off at 3/8 variables so as not to withhold the sepsis management guidelines to under-classified patients.

Our study has several limitations. Due to the fact that only surgical patients have been enrolled in the present study, it has to be clarified whether the presented results hold also true for patients of an internal medicine ICU. Out of the general and inflammatory variables, altered mental status and plasma procalcitonin were not applied in the present study. Mental status is difficult to apply in analgosedated or intubated patients. Plasma procalcitonin values could not be taken in the statistical evaluation due to low numbers, since procalcitonin is not routinely measured in our ICU. Thus, especially the lack of procalcitonin is limiting our conclusions, since this parameter is considered as one of the most interesting biomarkers in this field. Thus, a statistical model, including procalcitonin, might have come to a different result. The impact of procalcitonin has to be clarified in the future.

Clinical consequences of misclassification of sepsis in critically ill surgical patients might concern diagnosis, inclusion in clinical studies, and therapy.

Regarding diagnosis, in the 2003 definitions, the group consensus concluded that few, if any, patients in the early stages of the inflammatory response to infection are diagnosed with sepsis using in the 1992 definitions via the four arbitrary criteria. Therefore, in the 2003 definitions, sepsis is defined as infection and presence of "some" of the general and inflammatory variables shown in Table 1. The 2003 definitions were constructed to detect more cases of sepsis than the 1992 definitions. This aim has been achieved [8]. Frequencies of severe sepsis and septic shock were higher and mortality rates lower within the same patient collective, when the 2003 definitions (≥ 2/8 variables) instead of the 1992 definitions (≥ 2/4 variables) were applied in the same collective of postoperative/posttraumatic patients [8]. Since facilitating a bedside diagnosis of sepsis had primacy over research entry criteria [3], due to the term "some", uncertainty remained, how many of the 2003 variables to use to assign sepsis to a patient. When defining sepsis with ≥ 2/8 of the 2003 variables in the present study, 105 out of the 507 cases were assigned to septic shock. When changing from 3/8 to 4/8 variables, the number of septic shock cases remarkably decreased (Table 2) whereas cases with disagreement profoundly increased (Table 3). Taken together, a cut-off of ≥ 3/8 of the 2003 variables should be used to assign sepsis in patients without missing severely ill patients in shock.

Concerning therapy, due to the 1992 definitions, physicians are used to a cut-off of 2/4 variables. Regarding therapy and applicability of the severe sepsis and septic shock management guidelines [4,5], the present study revealed a disagreement in 9% of cases with septic shock regarding all patients with infections (43 out of 507) when changing from a cut-off of 2/8 to 5/8 variables (Table 3). Defining sepsis using a cut-off of 5/8 instead of 2/8 variables might under-classify more than one third of the cases with septic shock (40/105 = 38%), with the consequence of not applying the "Surviving Sepsis Campaign" (SSC) guidelines [4,5] (Table 2). Mis-classification, i.e., both, under-classification and over-classification, may be harmful to patients, when therapy is withhold or patients are set at risk to experience undesirable effects. With optimal classification, the desirable effects of adherence to evidence based recommendations (beneficial health outcome, less burden on staff and patients, and cost savings) will outweigh the undesirable effects (harms, more burden and greater costs). Thus, it would be interesting to compare the benefits and downsides, the numbers needed to treat (NNTs), risk/benefit and cost/benefit ratios of the SSC management recommendations based on different cut-offs of the general and inflammatory variables in the 2003 definitions to assign sepsis.

To reduce the enormous heterogeneity in patients within the diagnostic categories of sepsis, precise and commonly used definitions of the diagnostic categories of sepsis, of severity of disease and organ dysfunctions, and risk stratification models are an inevitable prerequisite for comparability of study results and treatment recommendations. In the present study, we focused on the diagnostic category of sepsis (sepsis, severe sepsis, septic shock). The odds ratio of death of septic shock patients applying a cut-off of 1/8 up to 7/8 criteria ranged between 2.6 and 4.2, with overlapping 95% confidence intervals (Table 2). In addition to the diagnostic category of sepsis, comprehensive demographic data including severity scores, such as the SAPS II [6] or SAPS 3 [9], to assess severity of illness and to predict vital status at hospital discharge, will be mandatory. Moreover, predisposing factors as lined out in the 2003 sepsis definitions by the PIRO model (i.e., predisposition, infection/insult, response and organ dysfunction) as a staging system for sepsis will have to be incorporated to build a valid staging system for sepsis and prediction of mortality [3,10]. In a PIRO staging model for risk stratification, only patients with both, tachypnea and tachycardia, were at increased risk of death. The odds of death increased approximately 30% to 50% for each increase in one level per individual PIRO component [10]. In a modified PIRO (predisposition, injury/infection, response = organ dysfunction)/SAPS 3 score, prediction of mortality was excellent broken down by diagnostic categories of sepsis (infection, sepsis, severe sepsis, septic shock) underlying the 1992 sepsis definitions and the highest SOFA score values for each organ for severity of organ dysfunction, and the discrimination was better than with the SAPS 3 alone [11]. Thus, a combination of well-defined diagnostic categories of sepsis, PIRO staging systems and severity scores (SAPS 3) potentially may be superior for risk stratification in severe sepsis clinical trails.

In the future, it has to be clarified whether, in addition to clinical scores with unequivocal and clearly defined cut-offs, well defined immune parameters, such as distinct biomarker profiles, may improve diagnosis of infection and severity of disease, prediction of outcome, guidance and success of therapeutic interventions [3,12,13].

## Conclusions

The present study reveals that the cut-off for general and inflammatory variables to classify patients as sepsis profoundly influences frequency and mortality rates of septic shock. Usage of a cut-off of greater than 3/8 variables to assess sepsis may not detect, and, thus, under-classify more than 10% of patients with shock at high risk of death resulting in delayed or lack of timely and focused critical care management. Thus, the present study underlines the need for a widespread use of a commonly accepted number of general and inflammatory criteria to classify sepsis to facilitate comparability, diagnosis, treatment recommendations, and enrolment strategies for clinical trials of critically ill surgical patients. We suggest to use a cut-off of ≥ 3/8 criteria within the 2003 definitions to assess sepsis to yield an optimal balance between benefits and downsides regarding consecutive management guidelines.

## Key messages

• Applying 1/8 up to 8/8 general and inflammatory variables to define sepsis within the 2003 sepsis definitions in surgical patients resulted in reduced detection of septic shock cases.

• Within the same surgical patient collective, applying a cut-off of 1/8 up to 8/8 general and inflammatory variables resulted in an increase of the mortality rate up to a cut-off of 6/8 variables.

• Risk of death was elevated in those surgical patients classified to be in septic shock with 1/8 up to 8/8 variables compared to those classified without shock (OR 2.6 - 4.2).

• As the under-classification rate dramatically increases for cut-off values larger than 3/8, we suggest to use ≥ 3/8 general and inflammatory variables to define sepsis with the 2003 definitions to yield the best balance between benefits and downsides regarding consecutive management guidelines.

## Abbreviations

ACCP/SCCM: American College of Chest Physicians/Society of Critical Care Medicine; 95% CI: 95% confidence interval; CI: confidence interval; ICU: intensive care unit; INR: International Normalized Ratio; MAP: mean arterial pressure, OR: odds ratio; n. a.: not applied; numbers needed to treat: NNT; PaO2/FiO2: partial pressure of arterial oxygen/fraction of inspired oxygen; SBP: systolic blood pressure; SAPSII: Simplified Acute Physiology Score; SCCM/ESICM/ACCP/ATS/SIS: Society of Critical Care Medicine/European Society of Critical Care Medicine/American College of Chest Physicians/American Thoracic Society/Surgical Infection Society; SD: standard deviation; SIRS: Systemic Inflammatory Response Syndrome; SOFA: Sequential Organ Failure Assessment Score; SSC: Surviving Sepsis Campaign; SvO_2_: mixed venous oxygen saturation; WBC: white blood cells.

## Competing interests

The authors declare that they have no competing interests.

## Authors' contributions

MW, MHL, MK, BH and MS participated in study conception, study design, data analysis, interpretation and drafting of the manuscript. MW, MH and MN participated in data acquisition. MT and MW participated in programming the computer-assisted scoring systems and data base, data analysis and interpretation of the manuscript. All authors read and approved the final manuscript.

## Pre-publication history

The pre-publication history for this paper can be accessed here:

http://www.biomedcentral.com/1471-2253/10/22/prepub
